# Supplementary material for: Content-rich biological network constructed by mining PubMed abstracts
Source: BMC Bioinformatics. 2004 Oct 8;5:147. doi: 10.1186/1471-2105-5-147 (PMC528731; doi:10.1186/1471-2105-5-147)
Supplement: Additional File 2 — The original results of the above study (non-essential files are deleted to keep the file size under the limit set by BMC bioinformatics). [file 1471-2105-5-147-S2.bz2 › chilibotAdditionalFile2/dip10/206ID8749394E701/html/N_SUH.html]

 


 **N** and **SUH** 
  
Found 7 abstracts in PubMed, retrieved 7.  
 

 What does Google say? 
 PDF only 
| .edu only 

---

- Int J Radiat Oncol Biol Phys, 2001   **Treatment results and prognostic factors of advanced T3 4 laryngeal carcinoma the University of California, San Francisco UCSF and Stanford University Hospital **SUH** experience.** .
  PURPOSE To review the UCSF **SUH** experience in the treatment of advanced T3 4 laryngeal carcinoma and to evaluate the different factors affecting locoregional control and survival.
  METHODS AND MATERIALS We reviewed the records of 223 patients treated for T3 4 squamous cell carcinoma of the larynx between October 957, and December 999.
  There were 187 men and 36 women, with a median age of 60 years range, 28 85 years .
  The primary site was glottic in 122 and supraglottic in 101 patients.
  We retrospectively staged the patients according to the 1997 AJCC staging system.
  One hundred and twenty seven patients had T3 lesions, and 96 had T4 lesions.
  132 had **N** 9 had **N** 5 had N2, and 17 had N3 disease.
  The overall stage was III in 93 and IV in 130 patients.
  Seventy nine patients had cartilage involvement, and 144 did not.
  Surgery was the primary treatment modality in 161 patients, of which 134 had postoperative radiotherapy RT , 11 had preoperative RT, 7 had surgery followed by RT and chemotherapy CT , and 9 had surgery alone.
  Forty one patients had RT alone, and 21 had CT with RT.
  Locoregional control LRC and overall survival OS were estimated using the Kaplan Meier method.
  Log rank statistics were employed to identify significant prognostic factors for OS and LRC.
  RESULTS The median follow up was 41 months range, 2 367 months for all patients and 78 months range, 6 332 months for alive patients.
  The LRC rate was 69% at 5 years and 68% at 10 years.
  Eighty four patients relapsed, of which 53 were locoregional failures.
  Significant prognostic factors for LRC on univariate analysis were primary site, **N** stage, overall stage, the lowest hemoglobin Hgb level during RT, and treatment modality.
  Favorable prognostic factors for LRC on multivariate analysis were lower **N** stage and primary surgery.
  The overall survival rate was 48% at 5 years and 34% at 10 years.
  Significant prognostic factors for OS on univariate analysis were primary site, age, overall stage, T stage, **N** stage, lowest Hgb level during RT, and treatment modality.
  Favorable prognostic factors for OS on multivariate analysis were lower **N** stage and higher Hgb level during RT.
  CONCLUSION Lower **N** stage was a favorable prognostic factor for LRC and OS.
  Hgb levels > or = 12.5 g dL during RT was a favorable prognostic factor for OS.
  Surgery was a favorable prognostic factor for LRC but did not impact on OS.
  Correcting the Hbg level before and during treatment should be investigated in future clinical trials as a way of improving therapeutic outcome in patients with advanced laryngeal carcinomas.

  - Gynecol Oncol< MedlineTA>Gynecol Oncol,   **Papers to Appear in Forthcoming Issues.** .
    Vulvar Squamous Carcinoma Prognostic Role of DNA Content, L.
    Mariani, L.
    Conti, G.
    Atlante, F.
    Sciarretta, M.
    Pozzi, M.
    Vercillo, V.
    Francavilla, L.
    De Franceschi, S.
    Rossi, R.
    Sindico, M.
    Atlante, and G.
    M.
    Gandolfo Primary Yolk Sac Tumor of the Endometrium A Case Report and Review of the Literature, Alain Spatz, Dorothée Bouron, Patricia Pautier, Damienne Castaigne, and Pierre Duvillard Hepatic Metastases from Carcinoma of the Uterine Cervix, Gwi Eon Kim, Sang Wook Lee, Chang Ok **Suh**, Tchan Kyu Park, Jae Wook Kim, Jong Taek Park, and Jae Uk Shim Mutation of the PTEN Tumor Suppressor Gene Is Not a Feature of Ovarian Cancers, G.
    Larry Maxwell, John I.
    Risinger, Betty Tong, Heather Shaw, J.
    Carl Barrett, Andrew Berchuck, and P.
    Andrew Futreal Laparoscopic Assistance for Extended Radicality of Vaginal Radical ysterectomy Description of a Technique, Marc Possover, Norman Krause, Rosemarie Kühne Heid, and Achim Schneider Panniculectomy A Useful Technique for the Obese Patient Undergoing Gynecological Surgery, Penelope I.
    Blomfield, Thao Le, David G.
    Allen, and Robert S.
    Planner Phase 2 Trial of Moderately High Dose Single Agent Mitoxantrone in Platinum and Paclitaxel Refractory Ovarian Cancer, Maurie Markman, Stuart M.
    Lichtman, Howard Homesley, Alexander Kennedy, Kenneth Webster, Scott Ernst, George Omura, and Jerome Belinson The Effect of Growth Hormone on the Bursting Strength of Ileal Anastomotic Segments in Radiation Injured Rat Bowel, Clifford R.
    Wheeless, Jr.
    , Vanna Zanagnolo, and Deborah Bowers Familial Papillary Serous Carcinoma of the Cervix, Peritoneum, and Ovary A Report of the First Case, Edward J.
    Kaplan, Thomas A.
    Caputo, Peter U.
    F.
    Shen, Robert I.
    Sassoon, and Robert A.
    Soslow Favorable Outcome of Ovarian Germ Cell Malignancies Treated with Cisplatin or Carboplastin Based Chemotherapy A Hellenic Cooperative Oncology Group Study, M.
    A.
    Dimopoulos, M.
    Papadopoulou, E.
    Andreopoulou, Chr.
    Papadimitriou, **N**.
    Pavlidis, G.
    Aravantinos, A.
    Aspropotamitis, A.
    Anagnostopoulos, G.
    Fountzilas, S.
    Michalas, and D.
    Pectacides Rare Case of Small Cell Carcinoma Arising from the Endometrium with Paraneoplastic Retinopathy, Isao Sekiguchi, Mitsuaki Suzuki, Ikuo Sato, Taeko Ohkawa, Hidetoshi Kawashima, and Syuichi Tsuchida Toward the Development of a Universal Grading System for Ovarian Epithelial Carcinoma.
    I.
    Prognostic Significance of Histopathologic Features Problems Involved in the Architectural Grading System, Yoshio Shimizu, Seiryu Kamoi, Satoshi Amada, Katsuhiko Atsumi, Futoshi Akiyama, and Steven G.
    Silverberg Ureteral Carcinoma presenting as a Complex Pelvic Mass in a Post Menopausal Patient, John H.
    Farley, Thomas H.
    Douglas, David G.
    Mcleod, and Charles R.
    Harrison Advanced Ovarian Carcinoma Presenting With a Cerebrovascular Accident, Mousa I.
    Shamonki, Robert C.
    Squatrito, and Charles E.
    Welander Primary Malignant Mixed Müllerian Tumor of the Vagina, Deborah Neesham, Paul Kerdemelidis, and James Scurry Copyright 1998 Academic Press.

    - J Bacteriol, 1996   **Zwittermicin A resistance gene from Bacillus cereus.** .
      Zwittermicin A is a novel aminopolyol antibiotic produced by Bacillus cereus that is active against diverse bacteria and lower eukaryotes L.A.
      Silo **Suh**, B.J.
      Lethbridge, S.J.
      Raffel, H.
      He, J.
      Clardy, and J.
      Handelsman, Appl.
      Environ.
      Microbiol.
      60 2023 203 994 .
      To identify a determinant for resistance to zwittermicin A, we constructed a genomic library from B. cereus UW85, which produces zwittermicin A, and screened transformants of Escherichia coli DH5alpha, which is sensitive to zwittermicin A, for resistance to zwittermicin A.
      Subcloning and mutagenesis defined a genetic locus, designated zmaR, on a 1.2 kb fragment of DNA that conferred zwittermicin A resistance on E. coli.
      A DNA fragment containing zmaR hybridized to a corresponding fragment of genomic DNA from B. cereus UW85.
      Corresponding fragments were not detected in mutants of B. cereus UW85 that were sensitive to zwittermicin A, and the plasmids carrying zmaR restored resistance to the zwittermicin A sensitive mutants, indicating that zmaR was deleted in the zwittermicin A sensitive mutants.
      zmaR is functional in B. cereus.
      Sequencing of the 1.2 kb fragment of DNA defined an open reading frame, designated ZmaR.
      Neither the nucleotide sequence nor the predicted protein sequence had significant similarity to sequences in existing databases.
      Cell extracts from an E. coli strain carrying zmaR contained a 43.5 kDa protein whose molecular mass and **N** terminal sequence matched those of the protein predicted by the zmaR sequence.
      The results demonstrate that we have isolated a gene, zmaR, that encodes a zwIttermicin A resistance determinant that is functional in both B. cereus and E. coli.

      - J Bacteriol, 1996   **A single amino acid change in Escherichia coli glycerol kinase abolishes glucose control of glycerol utilization in vivo.** .
        Escherichia coli glycerol kinase EC 2.7.1.30.
        ATP glycerol 3 phosphotransferase is a key element in glucose control of glycerol metabolism.
        Its catalytic activity is inhibited allosterically by the glycolytic intermediate, fructose biphosphate, and by the phosphotransferase system phosphocarrier protein, IIIGlc also known as IIAGlc .
        These inhibitors provide mechanisms by which glucose blocks glycerol utilization in vivo.
        We report here the cloning and sequencing of the glpK22 gene isolated from E.
        C.
        C.
        Lin strain 43, a strain that shows the loss of glucose control of glycerol utilization.
        DNA sequencing shows a single missense mutation that translates to the amino acid change Gly 304 to Ser G 304 S in glycerol kinase.
        The effects of this substitution on the functional and physical properties of the purified mutant enzyme were determined.
        Neither of the allosteric ligands inhibits it under conditions that produce strong inhibition of the wild type enzyme, which is sufficient to explain the phenotype of strain 43.
        However, IIIGlc activates the mutant enzyme, which could not be predicted from the phenotype.
        In the wild type enzyme, G 304 is located 1.3 nm from the active site and 2.5 nm from the IIIGlc binding site M.
        Feese, D.
        W.
        Pettigrew, **N**.
        D.
        Meadow, S.
        Roseman, and S.
        J.
        Remington, Proc.
        Natl.
        Acad.
        Sci.
        USA 91 3544 354 994 .
        It is located in the same region as amino acid substitutions in the related protein DnaK which alter its catalytic and regulatory properties and which are postulated to interfere with a domain closure motion A.
        S.
        Kamath Loeb, C.
        Z.
        Lu, W.
        C.
        **Suh**, M.
        A.
        Lonetto, and C.
        A.
        Gross, J.
        Biol.
        Chem.
        270 30051 3005 995 .
        The global effect of the G 304 S substitution on the conformation and catalytic and regulatory properties of glycerol kinase is consistent with a role for the domain closure motion in the molecular mechanism for glucose control of glycerol utilization.

        - Plant Physiol, 1995   **Tomato exo 1 >4 beta D galactanase.
          Isolation, changes during ripening in normal and mutant tomato fruit, and characterization of a related cDNA clone.** .
          An exo 1 >4 beta D galactanase was isolated from ripe tomato fruit Lycopersicon esculentum Mill.
          cv Ailsa Craig and cv Better Boy using anion exchange, gel filtration, and cation exchange chromatography.
          Sodium dodecyl sulfate polyacrylamide gel electrophoresis of the most active fraction revealed a predominant protein band at 75 kD and several minor bands.
          A 30 amino acid **N** terminal sequence from this 75 kD protein showed a high degree of homology with other recently identified beta galactosidase galactanase proteins from persimmon and apple fruits I.
          K.
          Kang, S.
          G.
          **Suh**, K.C.
          Gross, J.
          K.
          Byun 1994 Plant Physiol 105 975 979.
          G.S.
          Ross, T.
          Wegrzyn, E.A.
          MacRae, R.J.
          Redgwell 1994 Plant Physiol 106 521 528 and with the predicted polypeptide sequence encoded by the ethylene regulated SR12 gene in carnation K.G.
          Raghothama, K.A.
          Lawton, P.B.
          Goldsbrough, W.R.
          Woodson 1991 Plant Mol Biol 17 61 71 .
          The enzyme focused to a single band of beta galactosidase activity on an isoelectrofocusing gel at pH 9.8.
          The enzyme was specific for 1 >4 beta D galactan substrates with a pH optimum of 4.5.
          The only reaction product detected was monomeric galactose, indicating that the enzyme was an exo 1 >4 beta D galactanase.
          beta Galactanase activity increased at the onset of ripening in normal fruit, but no similar increase was detected in the nonripening mutants nor and rin.
          A tomato homolog pTom beta gal 1 was isolated using the SR12 cDNA clone from carnation as a probe.
          This clone showed 73% identify at the amino acid level with beta galactosidase related sequences from apple and asparagus and 66% identity with SR12.
          ABSTRACT TRUNCATED AT 250 WORDS

          - J Am Geriatr Soc, 1995   **Healthy young and old women differ in their trunk elevation and hip pivot motions when rising from supine to sitting.** .
            OBJECTIVE To describe the differences between healthy young and older women in regards to trunk elevation and hip pivot motions when rising from a supine to a seated position.
            DESIGN Cross sectional comparison.
            SETTING University laboratory.
            PARTICIPANTS Two groups of healthy female volunteers young adult female controls **n** = 22, mean age 23.5 years and community dwelling older female adults **n** = 17, mean age 73.8 years .
            MEASUREMENTS Subjects were videotaped as they performed three controlled bed mobility tasks, starting from a supine position 1 rising to a seated position at the edge of a firm plinth surface SS .
            and rising to a seated position without moving to the edge of the bed while either 2 using hands **SUH** or 3 not using hands SUNH .
            A series of movements involving the trunk were identified as subjects performed the SS task.
            RESULTS The older women were more likely to rotate and laterally flex their trunks, particularly in the later phases of the SS task.
            In addition, during the SS task, the older group was more likely to bear weight on their hip gluteal area, particularly in the later phases, and more likely to use a broad pivot base, consisting of the hip and the elbow.
            While all young and old performed the **SUH** task, less than half of the older group could complete the SUNH task.
            Moreover, the subgroup of older adults who could not complete the SUNH task may have accounted for much of the differences between the young and the old on the SS task.
            CONCLUSION Healthy young and older women differ in their ability to rise from a supine to sitting position, primarily in the strategies used to elevate the trunk and facilitate a pivot.
            Trunk flexion ability likely contributes to the age group differences noted in rising.
            These data provide the basis for a biomechanical analysis of the critical body segment motions and the strengths required to perform bed mobility tasks.

            - J Cell Biol, 1992   **Introduction of a gonadotropin receptor expression plasmid into immortalized granulosa cells leads to reconstitution of hormone dependent steroidogenesis.** .
              We have recently succeeded in immortalizing rat granulosa cells by co transfection with SV 40 DNA and the Ha ras oncogene.
              These cells lost their response to gonadotropins, but expressed the cytochrome P450scc mitochondrial system enzymes and produced progesterone and 20 alpha hydroxy 4 pregnan 3 one 20 alpha OH P upon cAMP stimulation **Suh**, B.
              S.
              , and A.
              Amsterdam.
              1990.
              Endocrinology.
              127 2489 2500.
              Hanukoglu, I.
              , B.
              S.
              **Suh**, S.
              Himmelhoch, and A.
              Amsterdam.
              1990.
              J.
              Cell Biol.
              111 1973 1981 .
              In an attempt to restore the steroidogenic response to gonadotropins in immortalized cells, lutropin choriogonadotropin LH CG R receptor expression plasmid was prepared by introducing the complete coding region of LH receptor cDNA McFarland, K.
              C.
              , R.
              Sprengel, H.
              S.
              Phillips, M.
              Köhler, **N**.
              Rosemblit, K.
              Nikolics, D.
              L.
              Segaloff, and P.
              H.
              Seeburg.
              1989.
              Science Wash.
              DC .
              245 494 499 into a SV 40 early promoter based eucaryotic expression vector.
              Granulosa cells from preovulatory follicles were transfected with this LH receptor expression plasmid, together with SV 40 DNA and the Ha ras oncogene.
              Cell lines obtained after this triple transfection accumulated cAMP in a dose dependent manner in response to hCG.
              Moreover, they produced progesterone and 20 alpha OH P upon hCG stimulation with an ED50 of 125 pM and 75 pM, respectively, which is within the physiological range.
              Concomitantly with hCG induced differentiation, inhibition of cell proliferation was evident following stimulation with hormone concentrations as low as 40 pM.
              The number of hCG receptor sites per cell after numerous passages and several freezing and thawing cycles was 1.9 x 10 4 , they showed a Kd of 180 pM.
              Stimulation with hCG induced pronounced morphological and biochemical changes in these cells including formation of mitochondrial located adrenodoxin, a marker enzyme for enhanced steroidogenesis.
              These findings make possible the expression in immortalized granulosa cells, of selectively mutated receptor molecules which preserve their steroidogenic potential, thereby opening the way to analysis of structure function relationships of the receptor molecule.
